# A Mathematical Model to Assess the Effect of Residual Positive Lymph Nodes on the Survival of Patients With Papillary Thyroid Microcarcinoma

**DOI:** 10.3389/fonc.2022.855830

**Published:** 2022-06-30

**Authors:** Wen Liu, Xuejing Yan, Zhizhong Dong, Yanjun Su, Yunhai Ma, Jianming Zhang, Chang Diao, Jun Qian, Tao Ran, Ruochuan Cheng

**Affiliations:** ^1^ Department of Thyroid Surgery, Clinical Research Center for Thyroid Disease of Yunnan Province, The First Affiliated Hospital of Kunming Medical University, Kunming, China; ^2^ Department of Management of Chronic Noncommunicable Diseases, Yunnan Center for Disease Control and Prevention, Kunming, China; ^3^ Department of Gastroenterology and Hepatology, The Second Affiliated Hospital, Chongqing Medical University, Chongqing, China

**Keywords:** papillary thyroid cancer, active surveillance, observation, overtreatment, lymph node metastasis, survival analysis

## Abstract

**Background:**

Active surveillance (AS) has been considered the first-line management for patients with clinical low-risk papillary thyroid microcarcinoma (PTMC) who often have lymph node micrometastasis (m-LNM) when diagnosed. The “low-risk” and “high prevalence of m-LNM” paradox is a potential barrier to the acceptance of AS for thyroid cancer by both surgeons and patients.

**Methods:**

Patients diagnosed with PTMC who underwent thyroidectomy with at least one lymph node (LN) examined were identified from a tertiary center database (n = 5,399). A β-binomial distribution was used to estimate the probability of missing nodal disease as a function of the number of LNs examined. Overall survival (OS) probabilities of groups with adequate and inadequate numbers of LNs examined were estimated using the Kaplan–Meier method in the Surveillance, Epidemiology, and End Results (SEER) database (n = 15,340). A multivariable model with restricted cubic splines was also used to verify the association of OS with the number of LNs examined.

**Results:**

The risk of residual m-LNM (missed nodal disease) ranged from 31.3% to 10.0% if the number of LNs examined ranged from 1 and 7 in patients with PTMC. With 7 LNs examined serving as the cutoff value, the intergroup comparison showed that residual positive LNs did not affect OS across all patients and patients aged ≥55 years (P = 0.72 and P = 0.112, respectively). After adjusting for patient and clinical characteristics, the multivariate model also showed a slight effect of the number of LNs examined on OS (P = 0.69).

**Conclusions:**

Even with the high prevalence, OS is not significantly compromised by persistent m-LNM in the body of patients with low-risk PTMC. These findings suggest that the concerns of LNM should not be viewed as an obstacle to developing AS for thyroid cancer. For patients with PTMC who undergo surgery, prophylactic central LN dissection does not provide a survival benefit.

## Introduction

In recent decades, the incidence of thyroid cancer has continuously increased. Over one million people in 26 high-income and medium-income countries are estimated to have suffered from an overdiagnosis of thyroid cancer from 2008 to 2012 (1). Small thyroid cancers, especially papillary thyroid microcarcinoma (PTMC) ≤1 cm in size, account for up to 50% of detected cases (2–4).

Since the 1990s, management with active surveillance (AS), an alternative to immediate surgery, has consistently produced excellent outcomes in many cohorts (5–7). AS provides an ideal solution to curb the transition from overdiagnosis to overtreatment of small papillary thyroid cancer (PTC). However, AS has not been implemented in many countries, including China, which contributes to the largest number of overdiagnoses of thyroid cancer worldwide (1, 8). Concerns regarding latent metastatic lymph nodes (LNs) in the body are a potential barrier to acceptance of AS management by both surgeons and patients (9–11). Lymph node micrometastasis (m-LNM) is one of the common clinical characteristics of PTMC and occurs in 50.5% of patients who have similar clinical characteristics to patients undergoing AS, according to the report from Kuma Hospital (12). A paradox seems to exist between extremely high m-LNM rates and the observation of low-risk PTC.

In addition to the necessity of surgery, another controversy of PTMC is the management of central LNs during surgery. Due to the insufficient level of evidence in current studies evaluating the association of lymph node dissection (LND) and long-term survival, controversy exists extensively among scholars and guidelines. European and American guidelines suggest that only clinically visible LNM is associated with a poor prognosis, and prophylactic central lymph node dissection (CLND) is not recommended in patients with low-risk PTC (13–15). Due to the high prevalence of micrometastases, the practice guidelines of East Asian countries such as China and Japan recommend routine prophylactic (at least ipsilateral) CLND (16, 17). In the past 5 years, at least 350,000 patients with PTMC have undergone CLND in China according to estimates from the International Agency for Research on Cancer data (18). The discordance in the recommendations between guidelines has decreased overall adherence and created confusion among surgeons, which is associated with a compromised patient prognosis (19, 20).

Due to the long natural course of PTC, a randomized controlled trial (RCT) to confirm survival outcomes may not be practical (21). Therefore, new statistical models must be explored to verify the potential effect of m-LNM in the body on long-term survival outcomes under existing conditions. In the present study, we adapted a previous statistical model to assess the adequate number of central LNs and quantify the risk of residual m-LNM. The Surveillance, Epidemiology, and End Results (SEER) registry database was subsequently used to evaluate the effect of residual positive LNs on the survival of patients with PTMC.

## Materials and Methods

### Probabilistic Model of an Adequate Lymph Node Yield

A single central database of consecutive cases from 2007 to 2020 was used to establish a probabilistic model of the distribution of central LNs (22). Patients with pathologically diagnosed PTC ≤1 cm who underwent thyroidectomy and CLND with one or more LNs examined were included. Patients with distant metastases or a history of head-neck surgery were excluded. Only patients who underwent surgeries performed by high-volume surgeons (>100 thyroidectomies/year) were included in our probabilistic model to ensure an adequate extent of lymphadenectomy. We focused on evaluating an adequate number of CLND in patients with PTMC, and lateral LNs were not included in the model.

**Figure 1:**
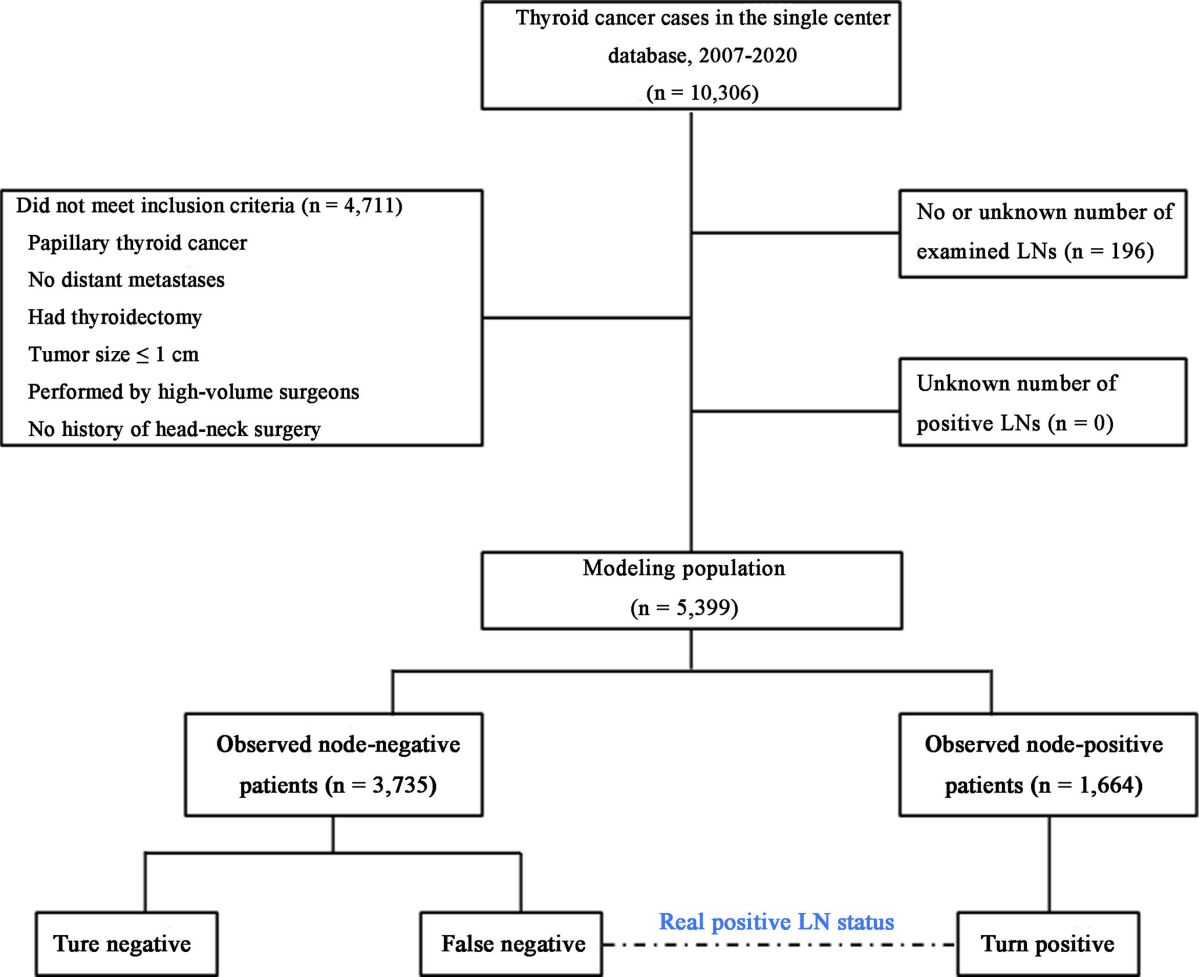
Flowchart of the modeling patient cohort. The patients were classified into three categories. LNs, lymph nodes.

As shown in **Figure 1**, presuming that no false positives are present in the pathological LN examination, the real LNM status is regarded as a true positive (TP) or false negative (FN), as calculated using the following equation:


(1) $$Prev.\;of\;real\;LNM\;=\;P\left(TP\right)+P\left(FN\right)\;=\;\frac{\#TP+\#FN}{\#TP+\#FN+\#TN}$$

A previous mathematical model described by Gönen et al. (23) was adapted and simplified to establish the probability model for estimating residual nodal disease in patients with PTMC. First, we constructed a model to estimate the distribution percentage of observed positive LNs in patients with at least two LNs examined. Second, we used a β-binomial distribution to calculate the prevalence of latent LN disease as a function of the number of LNs examined, which represents the probability of missing a positive node in observed node-negative patients (Equations 2, 3). A maximum likelihood approach was used to estimate the parameters (α and β) from individual patient data. *m* was used to indicate the number of examined LNs. This algorithm is based on the assumption that for a given patient, all LNs have an equal probability of being involved. Therefore, when deriving the probability of missed nodal disease in observed node-positive patients for each *m*, represented by *P(TPm)*, we averaged the calculated probabilities across patients. (See the *Supplementary Methods* for a detailed description of the derivation process.)


(2) $$P\left(Nm\right)\;=\;P\left(FNm\;+\;TNm\right)\;=\;\frac{Beta\;\left(\alpha ,\beta +m\right)}{Beta\;\left(\alpha ,\beta \right)}$$

(3) $$P\left(TPm\right)\;=\;1-\;P\left(FNm+TNm\right)\;=\;1-\frac{Beta\;\left(\alpha ,\beta +m\right)}{Beta\;\left(\alpha ,\beta \right)}$$

The number of real positive cases (including *#TPm* and *#FNm*) can be calculated by the ratio of *#TPm* to *P(TPm)*. Therefore, third, the probability of *P(TPm)*, allows us to calculate the number of FNs at each *m (#FNm)* by:


(4) $$\#FNm\;=\;\frac{P\left(FNm\;+\;TNm\right)*\#TPm}{P\left(TPm\right)}\;=\;\frac{P\left(FNm\;+\;TNm\right)*\#TPm}{1-P\left(FNm\;+\;TNm\right)}$$

Finally, based on the number of FNs we derived, the observed prevalence of LNM was corrected by summing over all *m*:


(5) $$Corrected\;Prev.\;of\;LNM\;=\;\frac{\sum m\left(\#TP+\#FN\right)}{\sum m\left(\#TP+\#FN+\#TN\right)}$$

### Residual Positive Lymph Nodes and Survival

The SEER database captures approximately 28% of the US population and contains complete long-term survival status records (24). We identified patients diagnosed with PTC between 2004 and 2015 using the International Classification of Disease for Oncology 3rd Edition (ICD-O-3) codes: 8050/3, 8260/3, 8340/3, 8341/3, 8342/3, and 8343/3.

Overall survival (OS) was analyzed only for patients with PTMC who underwent LND with observed negative LNs and were considered potential AS candidates. Patients with aggressive histologic variants were excluded. Patients with distant metastasis and a history of malignancy were excluded as well (**Supplementary Figure S1**). Regional LNs were defined as cervical and upper mediastinal LNs in the SEER records. We focused on evaluating the effects of possibly persistent positive and small LNs located in central compartments on OS of patients with PTMC (adequacy vs. inadequacy of LNs examined). However, direct variables to indicate the preoperative status of the LNs, such as size and location, were not available in the public database. Our analyses thus did not include patients with pathologic stage N1b disease because LND outside the central compartment was only performed for patients with PTMC presenting with clinically apparent lateral cervical LNM according to the recommendations of established guidelines. Estimates of OS probabilities were computed using the Kaplan–Meier method, and survival distributions were compared between groups using the log-rank test. As LNM exerts a greater effect on the survival of elderly patients, a subgroup analysis was also performed for patients aged ≥55 years.

### Number of Lymph Nodes Examined and Survival

We used another model to repeat the validation of the results of the survival analysis for all patients in the SEER registry to ensure the reliability of the study. Previous studies have documented robust correlations between continuous covariates (e.g., age and the number of metastatic LNs) and survival in patients with PTC. The potential confounders of continuous covariables were adjusted in a multivariable Cox proportional hazards (PH) regression model with restricted cubic spline (RCS) to examine the functional relationship between the number of LNs examined and OS. This relationship can be visualized using the flexible RCS model without prior knowledge of the form of the association (25, 26). We adjusted for the effects of patient sex, age, race, extrathyroidal extension, multifocality, extent of surgery, radioactive iodine (RAI) administration, and the number of positive LNs. Equally spaced knots were used, and the number of knots was determined by fitting models with 3–5 knots and selecting the model that minimized the Akaike information criterion (AIC) (25).

All survival analyses were performed using complete cases. The study was approved by the Ethics Committee of the First Affiliated Hospital of Kunming Medical University.

### Statistical Analysis

Analyses were independently performed and cross-validated by two authors (XY and TR) using R version 3.6.3 (R Foundation for Statistical Computing, Vienna, Austria) to ensure accuracy. The VGAM package was used to fit the α and β parameters of the β-binomial distribution using a maximum likelihood approach. Goodness-of-fit tests were used to evaluate the distribution of the model in which P > 0.05 was considered to satisfy the distribution. Finally, 95% confidence intervals (CIs) were calculated by creating 2,000 bootstrap samples and obtaining values at the 2.5% and 97.5% percentiles.

## Results

### Modeling the Patient Cohort

**Figure 2:**
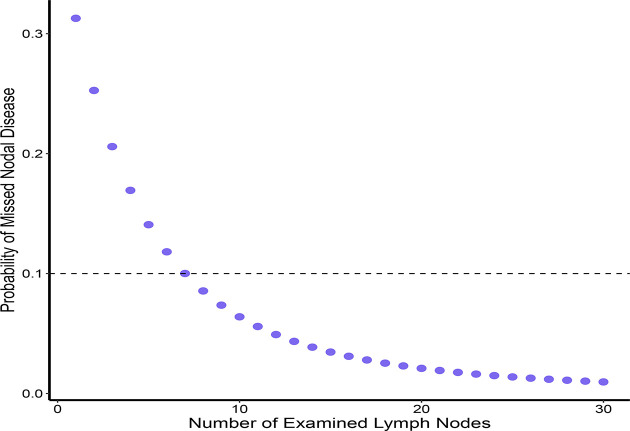
Probability of missed nodal disease as a function of the number of LNs examined.

Overall, 5,399 patients with PTMC met the inclusion criteria for our model (**Figure 1**). Of these patients, 1,664 (30.8%) had at least one positive LN (**Table 1**). Among patients with two or more LNs removed (n = 5,169), the probability distribution of the percentage of positive LNs was used to fit a β-binomial distribution. The distribution parameters were estimated to be α = 2.50 (95% CI: 2.17–2.94) and β = 6.21 (95% CI: 5.26–7.59) using the maximum likelihood approach. Thereafter, the probability of an FN as a function of the different numbers of LNs examined was estimated using this single set of parameters. When the number of LNs examined was one, two, three, four, five, six, and seven, the probability of an FN was estimated to be 31.3%, 25.3%, 20.6%, 16.9%, 14.1%, 11.8%, and 10.0%, respectively, in patients with PTMC. To rule out the risk of latent residual node positivity after central LND with 90% confidence, we needed to examine at least seven nodes in patients with PTMC (**Figure 2**). The sensitivity analysis of Hashimoto’s disease was basically consistent with the main analysis. When seven central LNs were examined, the probability of an FN was estimated to be 9.3% and 11.5% in patients with PTMC presenting with and without Hashimoto’s disease, respectively (**Supplementary Table S1**).

**Table 1:** Characteristics of the patients used to generate the model according to the LN status.

Characteristic	All Patients (%)(N = 5,399)	Node-negative (%)(N = 3,735)	Node-positive (%)(N = 1,664)	P value
Median age, years (IQR)	43 (36–51)	45 (38–52)	40 (33–48)	<0.001
Sex, Women	4,304 (79.7)	3,111 (83.3)	1,193 (71.7)	<0.001
Median LNs examined, No. (IQR)	7 (4–11)	6 (4–10)	9 (6–13)	<0.001
Clinical N0 stage	4,781 (88.6)	3,469 (92.9)	1,312 (78.8)	<0.001
Extrathyroidal extension	202 (3.7)	77 (2.1)	125 (7.5)	<0.001
thyroidectomy^†^				<0.001
Total thyroidectomy	3,153 (58.4)	2,075 (55.6)	1,078 (64.8)	
Lobectomy	2,246 (41.6)	1,660 (44.4)	586 (35.2)	
Central lymph node dissection^‡^				<0.001
Whole central compartment	3,200 (59.3)	2,062 (55.2)	1,138 (68.4)	
Ipsilateral central compartment	2,199 (40.7)	1,673 (44.8)	526 (31.6)	

^†^Total thyroidectomy included total/near total (3,149 cases) and subtotal thyroidectomy (4 cases). Lobectomy was defined as removal of a lobe with isthmus (2,067 cases), including additional contralateral lobotomy (171 cases) and isthmectomy alone (8 cases). ^‡^The superior, inferior, posterior, and external bounds of central LN dissection were the lower edge of the hyoid bone, sternal fossa, prevertebral fascia, and medial carotid artery sheath, respectively. Ipsilateral dissection included the unilateral central compartment and pretracheal LNs. IQR, interquartile range; LN, lymph node.

The overall population of 5,933 patients was pooled to estimate the real prevalence of LNM. The derived real prevalence of nodal disease (sum of the observed and estimated probabilities) was 38.9%.

### Effect of an Adequate Number of Lymph Nodes Examined on Survival

A total of 15,340 eligible patients with PTMC in the SEER database met the inclusion criteria, and 374 deaths were observed during a median follow-up of 63 (interquartile range 34–96) months. Of these, 2,890 patients were observed to have positive LNs. The LN yield was considerably lower in the SEER cohort than in our modeling cohort (median 2 vs. 7) (**Supplementary Table S2**). The corrected prevalence of LNM was substantially higher after FNs were calculated (18.8% to 33.6%) using the previously derived model.

**Figure 3:**
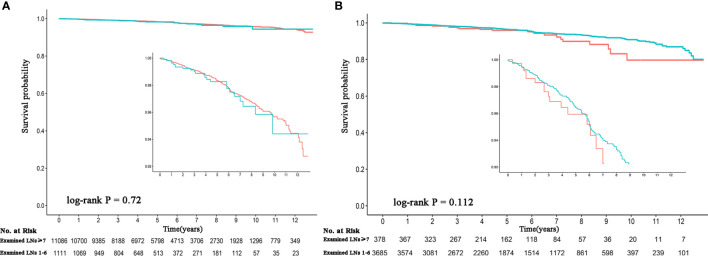
Comparison of the survival probability between an adequate and inadequate number of LNs examined among observed node-negative patients. **(A)** All patients. **(B)** Patients aged ≥55 years. The red line represents >7 LNs examined, and the green line represents 1–6 LNs examined. LNs, lymph nodes.

Patients with complete information (n = 12,197) were divided into an adequate group and an inadequate group (≥7 and 1–6 of LNs examined, respectively) to evaluate the effect of latent residual m-LNM on survival. With 7 LNs serving as the cutoff value, 90% of residual occult positive LNs could be eliminated in the adequate group. Patients with an inadequate number of LNs examined did not have compromised OS compared to patients with an adequate number of LNs examined (P = 0.72). The 5-year OS rate was 98.5% and the 10-year OS rate was 97.7% for patients with an adequate number of LNs examined, while the 5-year OS rate was 98.6% and the 10-year OS rate was 97.7% for patients with an inadequate number of LNs examined (**Figure 3A**). A subgroup analysis of patients aged ≥55 years showed consistent survival results (P = 0.112). The 5-year OS rate was 96.8% and the 10-year OS rate was 94.2% for patients with an adequate number of LNs examined, while the 5-year OS rate was 97.3% and the 10-year OS rate was 95.6% for patients with an inadequate number of LNs examined (**Figure 3B**).

### Number of Examined Lymph Nodes and Survival

**Figure 4:**
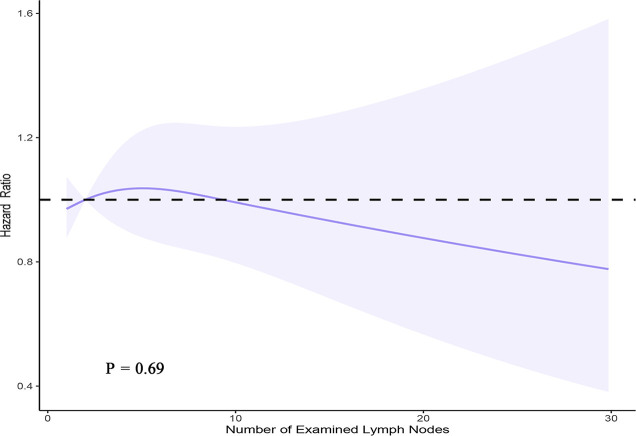
Smoothed RCS plot of the adjusted hazard ratio vs. the number of LNs examined using the SEER registry data. The estimates were adjusted for patient age, sex, race, extent of surgery, number of metastatic LNs, extrathyroidal extension, and RAI administration. Three knots were placed at one, two, and nine nodes (corresponding to the 10th, 50th, and 90th percentiles, respectively).

A PH regression model with RCS was used to verify the effect of the number of LNs examined on survival among 15,008 patients with complete information. The main variable (number of examined LNs) and continuous covariates (patient age and number of positive LNs) were modeled using a 3-knot RCS in the multivariable PH model (minimal AIC for the model with 3 knots). In the RCS model, fitting usually depends much more on the number of knots selected rather than the knot location. Therefore, knots were placed at fixed percentiles in this model (10, 50, and 90). Although a visual nonlinear association was observed, a significant overall association between the number of examined LNs and OS was not identified in the multivariable PH model with a 3-knot RCS (P = 0.69) (**Figure 4**).

## Discussion

Based on the mathematical model originally developed by Gönen et al. (23) and adapted by Robinson et al. (27) to evaluate the number of LNs examined, our study provides the first objective evaluation of the effect of residual m-LNM on the survival of patients with PTMC. The possible risk of persistent positive LNs was redefined in PTMC management. Our estimate suggests that at least seven LNs should be examined to ensure that the LN status is sufficiently assessed. The observed prevalence of m-LNM was substantially higher after correcting for FNs (18.8% to 33.6%) in the SEER registry data. However, based on the limited number of outcome events, no survival benefit was obtained from adequate CLND. The present study, despite the negative results, confirmed that latent residual m-LNM in patients with PTMC does not have the same prognostic effect as on other malignancies with LNM. We inferred that occult positive LNs do not cause a decrease in OS of patients with PTMC during AS. Moreover, unnecessary prophylactic CLND did not generate a survival benefit; in contrast, the risk of complications was increased.

In recent decades, statistical models based on probability theory have been widely used in physics, biology, medicine, economics, and other increasingly emerging fields. Given the very high human and economic costs, conducting RCTs is extremely difficult, especially in PTMC, which has a very long natural course. Therefore, probabilistic statistical models can be used in clinical medical research to obtain clinical information that is difficult to evaluate by analyzing retrospective data with traditional statistical methods. In the present study, assuming that a positive LN is a random event (A), the frequency of its occurrence in different numbers of LNs examined can be captured. With the increase in the sample size, the range of frequencies (P) of the random event (A) decreased for each LN yield. When a sufficient sample size is included, its occurrence frequencies (P) gradually tend to be stable and are considered the probability of the random event (A), which is calculated using the formula P (A) = p.

In traditional oncology studies, predictive models are often used to evaluate the potential probabilistic relationship and reveal the intrinsic relationships between clinical variables and outcomes. However, tumors are associated with a variety of epidemiological and clinical features, the interactions of which are not completely clear. The inclusion of all potential influencing factors in clinical studies is often impossible. Therefore, confounding factors detract from the prediction accuracy of traditional prediction models. In this probability theory-based study, only the distribution of positive LNs was observed without other variables. In theoretical mathematics, this probability is considered to exist objectively, and no confounding factors occur. Given the positive correlation between sample size and probability accuracy, a large single-center cohort with homogeneous clinical practice is particularly suitable for conducting research using a probabilistic model. This approach also assists in developing deeper studies for other cancers based on existing data.

For many years, LN evaluations in patients with PTC were mainly based on several indirect pieces of evidence. First, the American Thyroid Association (ATA) task force concluded that ≤5 m-LNM (<0.2 cm) did not carry the risk of recurrence in patients with clinically apparent metastases (clinical N1) (28). Second, larger LNs are usually evaluated with satisfactory accuracy using ultrasound combined with fine-needle aspiration cytology (FNAC), while the low sensitivity of these methods is mainly observed for smaller central LNs. Third, a study of 2,735 patients with low-risk PTMC showed that only 94 patients (4.0%) had more than 5 positive LNs (29). Fourth, a multivariate analysis showed that LNM significantly predicted a poor OS outcome for patients aged >45 years (30, 31). A comprehensive analysis of the National Cancer Database (NCDB) and SEER showed a small but significantly decreased OS for <45-year-old patients with LNM compared with those in the same age group without LNM and that incrementally more positive LNs up to six confers an additional mortality risk in this age group (25). Together, the effect of the presence of m-LNM on OS is small at best and probably most significant in older patients (13). However, these findings were based on data from positive LNs that were resected, and the survival risk of residual metastatic LNs in the body has not been adequately evaluated. Conceivably, the survival threat of resected positive LNs is not equal to the residual positive LNs in the body. These concerns have contributed to overtreatment of patients with low-risk PTMCs worldwide both in terms of nonsurgical management and in excessive LND during surgery.

Approximately two million new cases of thyroid cancer are estimated to have been diagnosed worldwide in the last 5 years (18). In the era of small thyroid cancer outbreaks, AS management is an alternative to immediate surgery that provides an ideal solution for the overdiagnosis and overtreatment of PTMC at present and curbs the transition from overdiagnosis to overtreatment. Increased tumor size and newly developed LNM are two indicators used to monitor AS (32). Since patients whose tumors are located away from the thyroid capsule are usually considered AS candidates, an increase in tumor size is accurately detected using ultrasound and used to guide delayed surgery in a timely manner. However, another indicator, the presence of LNM, has been less than satisfactory.

Due to the limitations of anatomical location, central LNM is detected with an extremely low sensitivity (6.7%–16.8%) by ultrasound (33, 34). The LNM rate is conceivably underestimated during AS, and the definition of “low-risk” seems to be an unreliable concept. Concerns about LNM are reflected in almost all practice guidelines, regardless of whether AS is recommended as the first-line management protocol for PTMC (13–15, 35, 36). The largest AS cohort to date from Kuma Hospital showed that the percentages of newly developed LN disease were as low as 1.5% in 1,295 patients with low-risk PTMC with a mean follow-up of 60 months (37). However, another study by the same authors conducted at the same institution as the aforementioned study reported that LNM was present in more than half (50.5%) of 594 surgical patients with similar clinical characteristics during the same period (12). An investigation of AS patients in Japan showed that “tumor metastasizing to another place” was the highest level of worry experienced by patients during AS (11). Many comments on the AS approach from China also expressed concerns regarding the high prevalence and long-term outcomes of m-LNM, which is the greatest obstacle to AS management in China. Therefore, this probabilistic model designed to evaluate the survival risk of residual m-LNM in the body will facilitate the development of AS management.

Concerns regarding m-LNM are also reflected in surgical cases of PTMC. Some surgeons tend to perform prophylactic central compartment dissection during the initial surgery to avoid the intuitive threats of residual positive LNs, even in the absence of any clinical evidence of LNM. This tendency is reflected in the SEER registry data included in the present study and many studies from Asia (29, 38, 39), although the ATA guidelines (2009 edition) stopped recommending prophylactic CLND for PTMC 13 years ago (40). The present study also provides objective evidence against the long-term controversial issue of prophylactic CLND for patients with low-risk PTMC in clinical practice, namely, whether residual m-LNM affects survival. Interestingly, our probabilistic model is based on a large amount of data on prophylactic CLND (88.6%). The frequencies of residual LNM reached 33.9% in the central compartment after initial surgical treatment in patients with intermediate- and high-risk differentiated thyroid carcinoma (41). The central LN yield reflects the thoroughness of the dissection, which is also related to the stratification of structural residual/recurrence risk after initial surgical treatment. A recent study of recurrent PTC showed that an LN yield fewer than 11 and positivity rate greater than 65% would be considered risk factors for future recurrences (42). Consistently, the construction of a probabilistic model enables an evaluation of the long-term prognostic effect of residual m-LNM under existing conditions. Clinicians can use the LN yield as supplementary information for the initial assessment of recurrence risk stratification to more accurately understand the persistence or recurrence risk, which is helpful to guide subsequent management decisions and individualized follow-up strategies for patients. The recurrence risk assessment, including LN yield, may reduce the frequency of RAI therapy and the magnitude of endocrine therapy in at least a subset of patients, which may help improve the quality of life of surviving patients. Moreover, 7 LNs is the cutoff value for an adequate number of LNs examined, but we do not advocate for berry-picking LND, which may increase the difficulty of the second operation and the risk of complications, while the patients may not experience a survival benefit. In fact, we agree that LND for PTMC should follow the principle of “all or nothing.”

Cancer concern is common among patients with PTMC under both AS management and immediate surgery. Anxiety in patients who undergo surgery may be related to surgical complications and recurrences. In contrast, patients who undergo AS can avoid invasive surgery and its complications, so anxiety is largely related to disease progression and metastasis, which are also a challenge on AS management. In an investigation for patients under AS, 37% of them were anxious about cancer progression, but 60% of the patients reported less anxiety during AS than when the cancer was found; AS was considered the most suitable management approach by 83% of the patients (11). Other studies also demonstrated that the mental health of the AS group was better than that of the surgery group. Levels of cancer concerns in patients under AS would decrease over time. It can be expected that, with the evidence from our present study, it will further relieve patients’ anxiety and enhance patient confidence to AS management (43, 44).

This study has several limitations. First, potential intrinsic confounders could not be eliminated from our retrospective study. Due to the long natural course of PTC, a randomized trial to confirm survival outcomes may not be practical (21). In some ways, the present study may be the best approach available to evaluate the effect of occult LNs on survival. Second, this probabilistic model is based on the necessary assumption that all LNs within a patient have the same probability of being involved, which is biologically untenable. LN yield, rather than the LN location or other specific factors, is the strongest factor for determining whether a positive LN would be missed. Therefore, the bias from this assumption is small at best in the probability calculations. Third, the modeling cohort was derived from patients with at least two LNs examined, which may slightly overestimate the risk of residual nodal disease. We further validated our model in patients with 1–30 LNs examined in both the single-center and SEER datasets that showed the reliability of our model (**Supplementary Table S3**). Fourth, unlike Robinson et al. (27), who used the NCDB to establish a model of occult LN disease in patients with PTC, potential selection bias exists when using data from a single-center database; therefore, our established probabilistic model may require multicenter verification. However, the number of LNs examined was relatively lower in the model by Robinson et al. (27) than in our PTMC model (median of 4 vs. 7), which may be due to differences in the thoroughness of LND among various clinical practices in the large database. Patients treated at academic facilities had approximately double the odds of having an adequate LN yield compared those treated at community centers [odds ratio (OR) 1.94 (95% CI 1.55–2.41), P < 0.001] (45). The estimates obtained from our model are based on procedures performed by high-volume surgeons, which may be more likely to reflect the real status of the LNs. Additionally, the preoperative information is not available in the large databases; by comparison, 88.6% of patients had clinical N0 stage disease in our cohort and were considered candidates for AS. Fifth, the number of events, especially for disease-specific mortality, was small in all databases. Therefore, survival analyses are more appropriately performed with data from large databases with long-term follow-up rather than from single-center databases. Our study provides a method for modeling based on single-center data combined with survival verification in a large database, which may minimize the interference of coding inaccuracies in large databases and increase the availability of data resources (46). Finally, the data from SEER are unable to assess recurrence, which is also an important disease-related outcome. We also look forward to evaluating disease-free survival outcomes in patients with PTMC using other databases that include recurrence information.

## Conclusions

To the best of our knowledge, we are the first to evaluate the survival risk of residual m-LNM in the body using a mathematical model of the number of examined LNs in patients with PTMC. Concerns of LNM should not necessarily be viewed as prohibitive to AS management. Prophylactic CLND is performed and does not provide survival benefits for patients with PTMC. These findings are helpful to decrease overtreatment in patients with thyroid cancer.

## Data Availability Statement

Publicly available datasets were analyzed in this study. These data can be found here: SEER database.

## Ethics Statement

The study was approved by the Ethics Committee of the First Affiliated Hospital of Kunming Medical University. The patients/participants in modeling cohort provided their written informed consent to participate in this study.

## Author Contributions

WL conceived the initial idea, analyzed the data, and wrote the paper. XY and TR performed the statistical analyses. All authors contributed to the study design, commented on drafts, and performed revisions. All authors contributed to the article and approved the submitted version.

## Funding

This work was supported by grants from the Construction Project of Clinical Research Centre of General Surgical Disease in Yunnan Province (2X2019-03-03) and the “Ten Thousand People Plan” of Yunnan Province–Medical Experts Project (RLCRC20210412).

## Conflict of Interest

The authors declare that the research was conducted in the absence of any commercial or financial relationships that could be construed as a potential conflict of interest.

## Publisher’s Note

All claims expressed in this article are solely those of the authors and do not necessarily represent those of their affiliated organizations, or those of the publisher, the editors and the reviewers. Any product that may be evaluated in this article, or claim that may be made by its manufacturer, is not guaranteed or endorsed by the publisher.
