# Supplementary material for: A Mathematical Model to Assess the Effect of Residual Positive Lymph Nodes on the Survival of Patients With Papillary Thyroid Microcarcinoma
Source: Front Oncol. 2022 Jun 30;12:855830. doi: 10.3389/fonc.2022.855830 (PMC9279734; doi:10.3389/fonc.2022.855830)
Supplement: Supplementary file 1 [file DataSheet_1.docx]

Supplementary Material

**
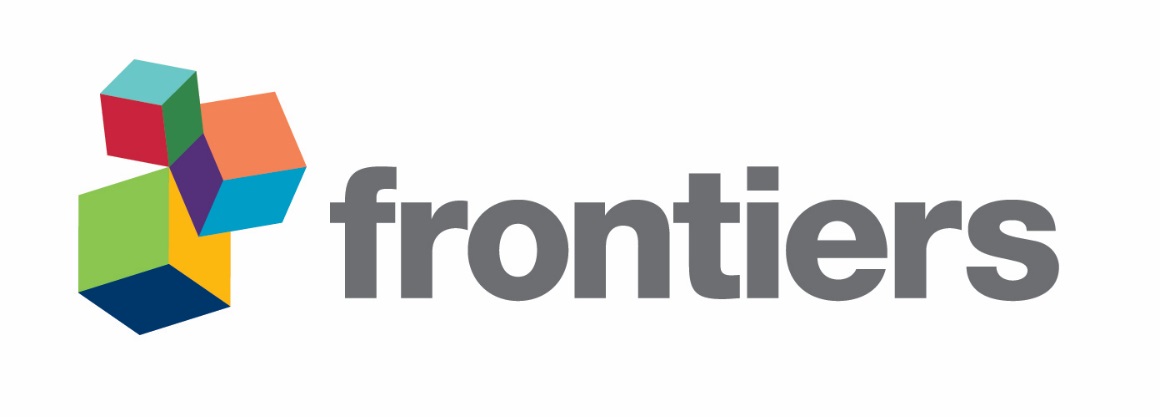
**

**Supplementary Methods**

**Procedure used to derive the mathematical model**

The model was constructed to estimate the distribution percentage of **observed positive LNs** in patients with at least two LNs examined. According to the hypothesis, no false positives are present in the pathological LN examination, and the observed positive LN status is regarded as a true positive (TP). In this case, *m* was used to indicate the number of examined LNs, and the probability of *k* positive nodes was calculated using the following equation:

$$P\left( k \right)=c_{m}^{k}\frac{Beta\left( k+\alpha,m-k+\beta\right)}{Beta (\alpha, \beta)}$$

where *k*=0, i.e., there are no positive nodes. We used a β-binomial distribution to calculate the prevalence of latent LN disease as a function of LNs examined, which represents the probability of missing a positive node in observed node-negative patients [*P(Nm)*]:

$$P\left( Nm \right)=P\left( FNm+TNm \right)=\frac{Beta (\alpha，\beta+m)}{Beta (\alpha, \beta)}$$

Therefore, the probability of observed positive LN, *P(TPm)*, was calculated using the following equation:

$$P\left( TPm \right)=1-P\left( FNm+TNm \right)=1-\frac{Beta (\alpha，\beta+m)}{Beta (\alpha, \beta)}$$

The number of real positive cases (*#TPm* and *#FNm*) for each *m* was calculated as follows:

$$\#TPm+\#FNm=\frac{\#TPm}{P\left( TPm \right)}=\#TPm/[1-\frac{Beta \left( \alpha，\beta+m \right)}{Beta \left( \alpha, \beta\right)}]$$

Finally, we calculated the number of FNs at each *m* (#FN*m*) using the following equation:

$$\#FNm=\frac{P\left( FNm+TNm \right) * \#TPm}{P\left( TPm \right)}=\frac{P\left( FNm+TNm \right) * \#TPm}{1 -P(FNm+TNm)}$$

Based on the number of FNs we derived, the observed prevalence of LNM was corrected by summing over all *m*.

$Corrected Prev. of LNM=\frac{\sum m(\#TP+\#FN)}{\sum m(\#TP+\#FN+\#TN)}$ (5)

**Supplementary Figure 1. Flow chart of the verified patient cohort.**


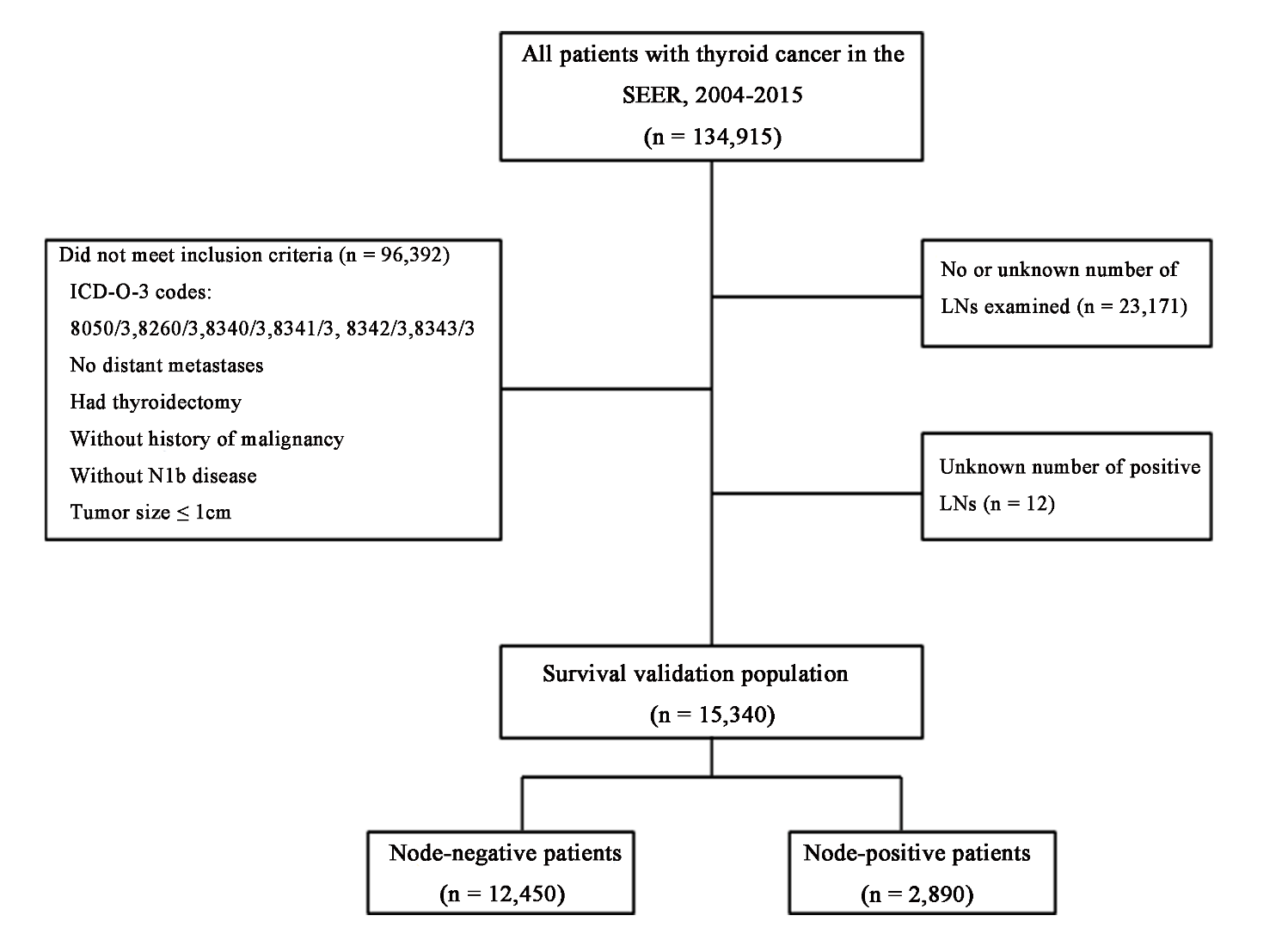


**Supplementary Table 1** Probability of FNs for LN metastasis based on the number of LNs examined in patients with PTMC

| Number of LNs examined | All patients (*n*=5150) | Without HT (*n*=3653) | With HT (*n*=1497) | Number of LNs examined | All patients (*n*=5150) | Without HT (*n*=3653) | With HT (*n*=1497) |
| --- | --- | --- | --- | --- | --- | --- | --- |
| 1 | 31.3 | 31.1 | 29.5 | 11 | 5.6 | 5.1 | 7.1 |
| 2 | 25.3 | 24.7 | 24.7 | 12 | 4.9 | 4.4 | 6.3 |
| 3 | 20.6 | 19.8 | 20.9 | 13 | 4.3 | 3.9 | 5.7 |
| 4 | 16.9 | 16.1 | 17.7 | 14 | 3.9 | 3.5 | 5.2 |
| 5 | 14.1 | 13.3 | 15.2 | 15 | 3.5 | 3.1 | 4.7 |
| 6 | 11.8 | 11.0 | 13.2 | 16 | 3.1 | 2.8 | 4.3 |
| 7 | 10.0 | 9.3 | 11.5 | 17 | 2.8 | 2.5 | 3.9 |
| 8 | 8.6 | 7.9 | 10.1 | 18 | 2.5 | 2.3 | 3.6 |
| 9 | 7.4 | 6.7 | 8.9 | 19 | 2.3 | 2.1 | 3.3 |
| 10 | 6.4 | 5.8 | 7.9 | 20 | 2.1 | 1.9 | 3.1 |

Only patients in whom at least two LNs were examined were included. Data regarding HT were unknown for 24 patients.

HT, Hashimoto’s disease; LN, lymph node

**Supplementary Table 2. Characteristics of patients in the SEER registry according to the observed LN status.**

| Characteristic | All Patients (%) (N=15340) | Node-negative (%) (N=12450) | Node-positive (%) (N=2890) | P value |
| --- | --- | --- | --- | --- |
| Sex, female | 12991 (84.7) | 10793 (86.7) | 2198 (76.1) | < 0.001 |
| Median age, years (IQR) | 48 (38–57) | 48 (39–58) | 44 (35–54) | < 0.001 |
| Median number of LNs examined, No. (IQR) | 2 (1–4) | 2 (1–4) | 2 (1–4) | < 0.001 |
| Race |  |  |  | < 0.001 |
| White | 13100 (85.4) | 10667 (85.7) | 2433 (84.2) |  |
| Black | 539 (3.5) | 473 (3.8) | 66 (2.3) |  |
| Other | 1475 (9.6) | 1133 (9.1) | 342 (11.8) |  |
| Extrathyroidal extension |  |  |  | < 0.001 |
| Yes | 1193 (7.8) | 641 (5.1) | 552 (19.1) |  |
| No | 14134 (92.1) | 11800 (94.8) | 2334 (80.8) |  |
| Multifocality |  |  |  | < 0.001 |
| Yes | 6037 (39.4) | 4614 (37.1) | 1423 (49.2) |  |
| No | 8450 (55.1) | 7318 (58.8) | 1132 (39.2) |  |
| Extent of surgery |  |  |  | < 0.001 |
| Total thyroidectomy | 13102 (85.4) | 10366 (83.3) | 2736 (94.7) |  |
| Lobectomy | 2152 (14.0) | 2024 (16.3) | 128 (4.4) |  |
| RAI administration | 4886 (31.9) | 2907 (23.3) | 1979 (68.5) | < 0.001 |

Patients with an unknown status and missing data were excluded from the statistical analyses.

Data for race were unknown for 226 patients (1.5%). Data for extrathyroidal extension were unknown for 13 patients (0.1%). Data for multifocality were unknown for 853 patients (5.5%). Data for the extent of surgery were unknown for 86 patients (0.6%).

**Supplementary Table 3 Validation of the model in patients with 1 to 30 LNs examined.**

| Number of LNs examined | FN predictive value in a single-center database (predicted negative cases) | Real negative cases in a single-center database | FN predictive value in the SEER database (predicted negative cases) | Real negative cases in the SEER database |
| --- | --- | --- | --- | --- |
| 1 | 62.15007 | 205 | 1255.431 | 5155 |
| 2 | 59.67609 | 316 | 462.7712 | 2619 |
| 3 | 66.99867 | 371 | 211.9346 | 1403 |
| 4 | 53.03105 | 355 | 108.4086 | 887 |
| 5 | 43.14751 | 332 | 69.72637 | 600 |
| 6 | 34.52193 | 341 | 40.3641 | 375 |
| 7 | 27.63088 | 291 | 31.00564 | 284 |
| 8 | 24.19719 | 260 | 19.7353 | 218 |
| 9 | 17.21742 | 217 | 17.21742 | 173 |
| 10 | 12.10049 | 208 | 9.225129 | 144 |
| 11 | 8.786473 | 145 | 6.436602 | 111 |
| 12 | 8.190709 | 121 | 5.548545 | 80 |
| 13 | 5.593923 | 110 | 4.904261 | 69 |
| 14 | 4.032861 | 90 | 3.226289 | 53 |
| 15 | 2.375115 | 73 | 2.434492 | 41 |
| 16 | 2.533833 | 57 | 1.266917 | 33 |
| 17 | 1.88787 | 57 | 1.510296 | 41 |
| 18 | 1.230003 | 30 | 1.314831 | 35 |
| 19 | 1.033911 | 34 | 0.804153 | 17 |
| 20 | 0.694381 | 34 | 0.694381 | 18 |
| 21 | 0.821625 | 12 | 0.442413 | 11 |
| 22 | 0.548448 | 13 | 0.606179 | 15 |
| 23 | 0.211633 | 10 | 0.396812 | 9 |
| 24 | 0.291819 | 7 | 0.218864 | 11 |
| 25 | 0.224183 | 7 | 0.26902 | 10 |
| 26 | 0.207216 | 10 | 0.331546 | 5 |
| 27 | 0.096004 | 10 | 0.192008 | 2 |
| 28 | 0.053499 | 1 | 0.178329 | 1 |
| 29 | 0.066394 | 5 | 0.082993 | 3 |
| 30 | 0.046444 | 2 | 0.046444 | 2 |
